# Methylxanthine Treatment in Patients Hospitalized for Acute Exacerbation of Chronic Obstructive Pulmonary Disease in China: A Real-World Study Using Propensity Score Matching Analysis

**DOI:** 10.3389/fphar.2022.802123

**Published:** 2022-01-25

**Authors:** Zijie Zhan, Yiming Ma, Ke Huang, Chen Liang, Xihua Mao, Yaowen Zhang, Xiaoxia Ren, Jieping Lei, Yan Chen, Ting Yang, Chen Wang

**Affiliations:** ^1^ Department of Pulmonary and Critical Care Medicine, The Second Xiangya Hospital, Central South University, Changsha, China; ^2^ Department of Pulmonary and Critical Care Medicine, China-Japan Friendship Hospital, Beijing, China; ^3^ National Clinical Research Center for Respiratory Disease, Beijing, China; ^4^ Institute of Respiratory Medicine, Chinese Academy of Medical Science, Beijing, China; ^5^ Chinese Alliance for Respiratory Diseases in Primary Care, Beijing, China; ^6^ Chinese Academy of Medical Sciences and Peking Union Medical College, Beijing, China

**Keywords:** chronic obstructive pulmonary disease, acute exacerbation, hospitalization, methylxanthine, length of stay

## Abstract

**Background:** Although medical guidelines discourage the use of methylxanthines in patients with acute exacerbation of chronic obstructive pulmonary disease (AECOPD), they are still widely used in clinical practice. This study investigated the real-world use of methylxanthines in the management of AECOPD.

**Methods:** Patient data from the Acute exacerbation of Chronic obstructive pulmonary disease Using REgistry data (ACURE, NCT02657525) study database were screened. Enrolled patients were divided into treatment and control groups. Propensity score (PS) matching and Cox regression analyses were used to minimize confounding factors and determine the association between methylxanthine treatment and the length of stay (LOS).

**Results:** Among the 2088 eligible patients, 1,563 (74.9%) were in the methylxanthine treatment group. Patients treated with methylxanthines had more severe respiratory symptoms and worse lung function than those in the control group. Doxophylline was the most commonly used methylxanthine in both secondary and tertiary hospitals. After PS matching, 966 patients were equally divided into two groups. The LOS of patients in the two groups was similar [median: 8 days, interquartile range (IQR): 7–11 days, *p* = 0.730]. Patients in the treatment group (median: 8, IQR: 4–12) had a more significant decrease in the COPD Assessment Test score from admission to discharge than those in the control group (median: 6, IQR: 2–10, *p* < 0.001). Among all matched patients, the LOS was not significantly associated with methylxanthine treatment [adjusted hazard ratio (HR): 1.02, 95% confidence intervals (CIs): 0.89–1.16]. However, in the subgroup analysis, methylxanthines were significantly associated with a short LOS in patients with blood eosinophil count >4% (adjusted HR: 1.56, 95% CIs: 1.12–2.17).

**Conclusion:** This study revealed that methylxanthines, especially doxophylline, are widely used in China. Methylxanthines were effective in improving symptoms in AECOPD patients. Higher blood eosinophil count may be associated with a better efficacy of methylxanthine treatment.

## Introduction

Chronic obstructive pulmonary disease (COPD) is characterized by chronic respiratory symptoms and persistently inadequate airflow. COPD is highly prevalent worldwide and is associated with high disability and mortality according to the Global Burden of Disease study, making it a major public health challenge. In 2017, 299.4 million COPD cases and 3.2 million COPD-related deaths were reported worldwide (GBD 2017 Causes of Death Collaborators, 2018; GBD 2017 Disease and Injury Incidence and Prevalence Collaborators, 2018). In China, 99.9 million cases were reported, and COPD was the third leading cause of death in 2015 (Zhou et al., 2016; Wang et al., 2018). Acute exacerbation of COPD (AECOPD) is a significant event in the course of the disease, and develops in patients approximately 0.5–3.5 times annually. Further, AECOPD is associated with an accelerated decline of lung function, worse quality of life, and increased risk of mortality (Seemungal et al., 1998; Donaldson et al., 2002; Cai et al., 2014; Müllerova et al., 2015). Therefore, effective pharmacological treatments are required to minimize the impact of this condition on patients’ health and well-being. Methylxanthines, such as theophylline, have been used in the treatment of COPD and asthma for more than 80 years. They are known to promote bronchodilation and enhance inspiratory muscle function (Barnes, 2013). In addition, theophylline has an anti-inflammatory effect and can reverse corticosteroid resistance (Ito et al., 2002; Cosio et al., 2004). However, previous randomized control trials on methylxanthine treatment for AECOPD have indicated frequent side effects and drug interactions (Barr et al., 2003; Duffy et al., 2005). Based on these results, the Global Initiative for Chronic Obstructive Lung Disease (GOLD) does not recommend the use of methylxanthines in AECOPD patients. Similarly, the Chinese expert consensus recommended the use of methylxanthines only in selected patients who have an insufficient response to short-acting bronchodilators or in some severe AECOPD patients (Cai et al., 2014; Global, 2019).

However, methylxanthines, especially doxophylline, which is a methylxanthine derivative, are still widely used for AECOPD in actual clinical settings in China. It has been reported that doxophylline has better efficacy and safety than other types of methylxanthines, such as theophylline and aminophylline (Cazzola et al., 2018). However, the current use of methylxanthines in China and their efficacy and safety in real-world settings have not been described.

In this study, we first described important aspects of current usage of methylxanthines in hospitalized AECOPD patients in China and subsequently analyzed the efficacy of methylxanthine use in such settings.

## Methods

### Data Source

Data for this multicenter retrospective cohort study were obtained from the Acute exacerbation of Chronic obstructive pulmonary disease Using REgistry (ACURE, NCT02657525) database (Pei et al., 2020). The ACURE study is an ongoing, nationwide, multicenter, prospective study that is investigating the demographic characteristics, clinical features, diagnosis, treatments, disease prognosis, and economic costs associated with hospitalized patients with exacerbations of COPD in real-world settings. Additional details of the ACURE study have been described elsewhere (Pei et al., 2020). The study discussed herein was approved by the Ethics Committee of the China-Japan Friendship Hospital (2015-88) and was conducted in accordance with the ethical standards stated in the Helsinki Declaration.

### Patient Selection

We used data from the ACURE database starting from January 2018 to December 2019 for patients with AECOPD hospitalized at 163 secondary or tertiary hospital sites. The inclusion criteria for patients were the following: patients who 1) were not less than 40 years old; 2) had spirometry test results and met the diagnostic criteria according to the 2020 GOLD report (Global, 2019), which is a ratio of the post-bronchodilator forced expiratory volume in one second (FEV1) and forced vital capacity (FVC) of less than 0.7; and 3) were hospitalized due to AECOPD. Patients were excluded if they: 1) refused or withdrew informed consent; 2) had enrolled in other interventional studies; 3) had a diagnosis of pneumonia or chronic respiratory disease other than COPD, including asthma, lung cancer, bronchiectasis, and pulmonary fibrosis; and 4) were allergic to methylxanthines.

### Measurements and Outcomes

We extracted baseline characteristics of enrolled patients including demographic data, hospital sites of admission, smoking history, body mass index (BMI), AECOPD symptoms at admission, comorbid disease (cardiovascular disease, cerebrovascular disease, and diabetes) history, scores of the modified British Medical Research Council (mMRC) questionnaire, and COPD Assessment Test (CAT) questionnaire at admission, COPD severity, hospitalization history in the previous year, spirometry test results, and blood eosinophil counts. Dyspnea in AECOPD patients was assessed using the mMRC questionnaire, and other symptoms were assessed using the PEACE questionnaire from the Effect of Carbocisteine on Acute Exacerbation of chronic obstructive Pulmonary disease study (Zheng et al., 2008). The severity of COPD was assessed according to the GOLD staging system given in the 2020 GOLD report (Global, 2019). A history of pharmacological treatments undertaken during hospitalization, including the use of methylxanthines, bronchodilators, and corticosteroids, were also extracted. We divided the enrolled patients into treatment and control groups based on the use of methylxanthines.

The primary outcome was the length of stay (LOS), and the secondary outcomes were a change in the CAT score from admission to discharge, total direct costs, intensive care unit (ICU) admission and mortality during hospitalization, the CAT score at the 30-days follow-up visit, all-cause readmissions, and AECOPD-related readmissions within 30 days after discharge. Total direct costs were converted to United States dollars using the average exchange rate in 2019 (1 United States dollar was equivalent to 6.90 Yuan) because most patients were enrolled in this year.

### Statistical Analysis

Continuous variables with normal distributions have been presented as a mean with standard deviation, whereas non-normally distributed continuous variables have been presented a median and interquartile range (IQR). Categorical variables have been presented as frequency and percentage. To compare variables in the two groups, Student’s *t*-test or the Wilcoxon rank sum test were used for continuous variables and the Chi-square test or Fisher’s exact test were used for categorical variables.

To reduce the effect of confounding factors in this non-experimental study, a propensity score (PS) matching was performed, using the “MathIt” package in R software (Brookhart et al., 2013; Zhang, 2017). We calculated the PS using multivariable logistic regression. Demographic characteristics, including the age, sex, and other baseline variables with a *p*-value <0.1 in univariable analysis were included in the model. In addition, we used the “nearest” method and a caliper equal to 0.05 to conduct the PS match with a 1:1 ratio.

We used the Cox proportional hazards model to estimate the hazard ratio (HR) between methylxanthine treatment and the LOS. The outcome of the event was defined as hospital discharge. Both univariate and multivariate analyses were performed. All factors with a *p*-value <0.05, following a univariable analysis, were included in the multivariable analysis. HRs > 1 represented early discharge and a short LOS, while HRs < 1 represented late discharge and a long LOS.

In addition, we performed a subgroup analysis to examine the association between methylxanthine treatment and the LOS in subsets of the population. Patients were divided into subgroups according to the age, sex, smoking history, severity of AECOPD symptoms (cough, sputum, dyspnea, and wheezing), CAT score, GOLD stage, hospitalization in the previous year, and blood eosinophil counts. Cough symptoms were considered to be light if patients had no cough or only morning cough, whereas severe cough symptoms were considered to be episodes of cough during the day or nearly continuous cough.

Statistical analyses were performed using the R software (version 4.0.2). All statistical tests were two-sided tests, with significance set at *p* < 0.05.

## Results

### Baseline Characteristics

**FIGURE 1:**
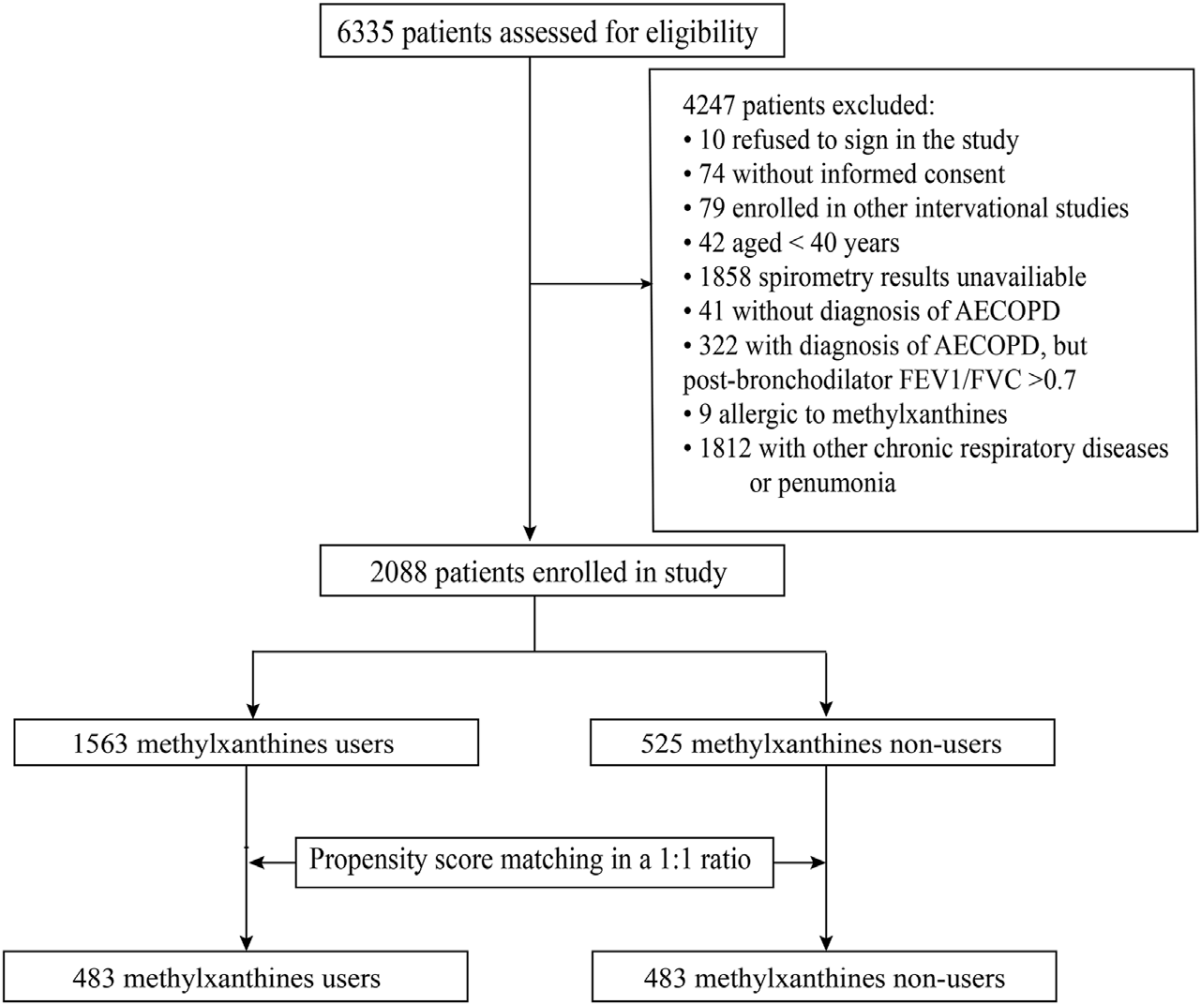
The flowchart of patient enrollment process. AECOPD, acute exacerbations of chronic obstructive pulmonary disease, FEV1/FVC, forced expiratory volume in one second/forced vital capacity.

A total of 2088 patients met all the eligibility criteria (Figure 1). Among them, 247 patients were from secondary hospitals and 1841 patients were from tertiary hospitals. The median age of the included patients was 70 (IQR: 64–76) years, and 1,679 (80.4%) patients were male. A total of 589 (28.2%) patients were current smokers, 913 (43.7%) were former smokers, and 586 (28.1%) were non-smokers. Among the included patients, 1,563 (74.9%) patients received methylxanthine treatment during hospitalization (treatment group) and 525 (25.1%) patients did not receive methylxanthine treatment (control group).

Demographic characteristics and clinical factors at admission of all enrolled patients before and after PS matching are given in Table 1, and treatment course during hospitalization has been summarized in Table 2.

**TABLE 1:** Baseline characteristics of hospitalized acute exacerbation of chronic obstructive pulmonary disease patients at admission.

Variables	Unmatched groups		*p*-Value	Matched groups		*p*-Value
	Treatment group (*n* = 1,563)	Control group (*n* = 525)		Treatment group (*n* = 483)	Control group (*n* = 483)	
Age (year)	69 (63–76)	70 (64–76)	0.245	70 (64–76)	70 (64–76)	0.953
Sex, male	1,262 (80.7)	417 (79.3)	0.512	385 (79.7)	387 (80.1)	0.872
Race, han	1,483 (94.9)	500 (95.2)	0.746	460 (95.2)	461 (95.4)	0.879
Smoking history			0.029*			0.718
Current smokers	451 (28.9)	138 (26.3)	-	123 (25.5)	133 (27.5)	-
Former smokers	697 (44.6)	216 (41.1)	-	206 (42.7)	205 (42.4)	-
Non smokers	415 (26.6)	171 (32.6)	-	154 (31.9)	145 (30.0)	-
BMI (kg/m^2^)	22.0 (19.5–24.4)	22.0 (19.4–24.7)	0.473	22.0 (19.6–24.6)	22.2 (19.3–24.8)	0.618
Symptoms						
Cough			0.002*			0.933
None	27 (1.7)	16 (3.0)	-	11 (2.3)	14 (2.9)	-
Morning only	576 (36.9)	233 (44.4)	-	207 (42.9)	205 (42.4)	-
Episodes during day	767 (49.1)	223 (42.5)	-	219 (45.3)	216 (44.7)	-
Nearly continuous	193 (9.2)	53 (10.1)	-	46 (9.5)	48 (9.9)	-
Sputum quantity			0.542			0.638
<10 ml	137 (8.8)	47 (9.0)	-	51 (10.6)	44 (9.1)	-
10–50 ml	789 (50.5)	278 (53.0)	-	251 (52.0)	255 (52.8)	-
50–100 ml	560 (35.8)	181 (34.5)	-	158 (32.7)	167 (34.6)	-
>100 ml	77 (3.7)	19 (3.6)	-	23 (4.8)	17 (3.5)	-
Wheezing	1,347 (86.2)	428 (81.5)	0.010*	405 (83.9)	398 (82.4)	0.548
Dyspnea (mMRC score)			<0.001*			0.720
0	13 (0.8)	14 (2.7)		7 (1.4)	10 (2.1)	-
1	205 (13.1)	81 (15.4)		64 (13.3)	74 (15.3)	-
2	477 (30.5)	163 (31.0)		166 (34.4)	150 (31.1)	-
3	581 (37.2)	205 (39.0)		185 (38.3)	188 (38.9)	-
4	287 (18.4)	62 (11.8)		61 (12.6)	61 (12.6)	-
CAT score	20 (15-25)	19 (15-23)	0.075	20 (15-24)	19 (15-23)	0.420
Comorbidities						
Cardiovascular disease	777 (49.7)	233 (44.4)	0.034*	212 (43.9)	218 (45.1)	0.698
Cerebrovascular disease	69 (4.4)	34 (6.5)	0.059	24 (5.0)	25 (5.2)	0.883
Diabetes	145 (9.3)	46 (8.8)	0.723	43 (8.9)	43 (8.9)	1.000
Previous year admission			0.079			0.914
None	769 (49.2)	272 (51.8)	-	251 (52.0)	244 (50.5)	-
Once	384 (24.6)	135 (25.7)	-	119 (24.6)	127 (26.3)	-
Twice	257 (16.4)	86 (16.4)	-	79 (16.4)	81 (16.8)	-
More than twice	153 (9.8)	32 (6.1)	-	34 (7.0)	31 (6.4)	-
Spirometry						
Post-dose FEV1 (l)	0.96 (0.72–1.34)	0.99 (0.74–1.45)	0.058	0.98 (0.73–1.38)	0.99 (0.74–1.44)	0.616
Post-dose FEV1/FVC	0.49 (0.41–0.58)	0.52 (0.43–0.60)	<0.001*	0.50 (0.42–0.59)	0.52 (0.43–0.60)	0.239
GOLD stage			<0.001*			0.634
I (mild)	98 (6.3)	63 (12.0)	-	47 (9.7)	47 (9.7)	-
II (moderate)	436 (27.9)	140 (26.7)	-	120 (24.8)	136 (28.2)	-
III (severe)	649 (41.5)	213 (40.6)	-	213 (44.1)	196 (40.6)	-
IV (very severe)	380 (24.3)	109 (20.8)	-	103 (21.3)	104 (21.5)	-
Eosinophil count			0.770			0.754
Eos ≤ 2% n (%)	912 (58.8)	305 (59.6)	-	282 (58.8)	282 (59.7)	-
Eos > 2% n (%)	638 (41.2)	207 (40.4)	-	198 (41.2)	190 (40.3)	-

Data are presented as n (%) or median (interquartile range). Abbreviations: BMI, body mass index; CAT, COPD assessment test; Eos, eosinophil; FEV1, forced expiratory volume in one second; FVC, forced vital capacity; mMRC, modified british medical research council.

**TABLE 2:** In-hospital treatment of acute exacerbation of chronic obstructive pulmonary disease patients with or without methylxanthines.

	Before PSM		*p*-Value	After PSM		*p*-Value
	Treatment group (*n* = 1,563)	Control group (*n* = 525)		Treatment group (*n* = 483)	Control group (*n* = 483)	
Short-bronchodilators						
SAMA	591 (37.8)	135 (25.7)	<0.001*	119 (24.6)	132 (27.3)	0.340
SABA	839 (53.7)	265 (50.5)	0.203	257 (53.2)	252 (52.2)	0.747
Antibiotics	1,419 (90.8)	416 (79.2)	<0.001*	421 (87.2)	400 (82.8)	0.059
Corticosteroids						
Inhaled CS	280 (17.9)	69 (13.1)	0.011 *	69 (14.3)	68 (14.1)	0.926
Oral CS	60 (3.8)	10 (1.9)	0.033 *	13 (2.7)	9 (1.9)	0.388
Transvenous CS	500 (32.0)	119 (22.7)	<0.001 *	121 (25.1)	118 (24.4)	0.823
Nebulized CS	965 (61.7)	227 (43.2)	<0.001 *	212 (43.9)	221 (45.8)	0.560
Methylxanthines						
Doxophylline	1,264 (80.9)	NA	-	384 (79.5)	NA	-
Aminophylline	153 (9.8)	NA	-	42 (8.7)	NA	-
Diprophylline	147 (9.4)	NA	-	46 (9.5)	NA	-
Theophylline	69 (4.4)	NA	-	26 (5.4)	NA	-

Data are presented as n (%) or median (interquartile range). Abbreviations: CS, corticosteroid; PSM, propensity score matching; SAMA, short-acting muscarinic antagonist; SABA, short-acting beta-antagonists; NA, not applicable.

**FIGURE 2:**
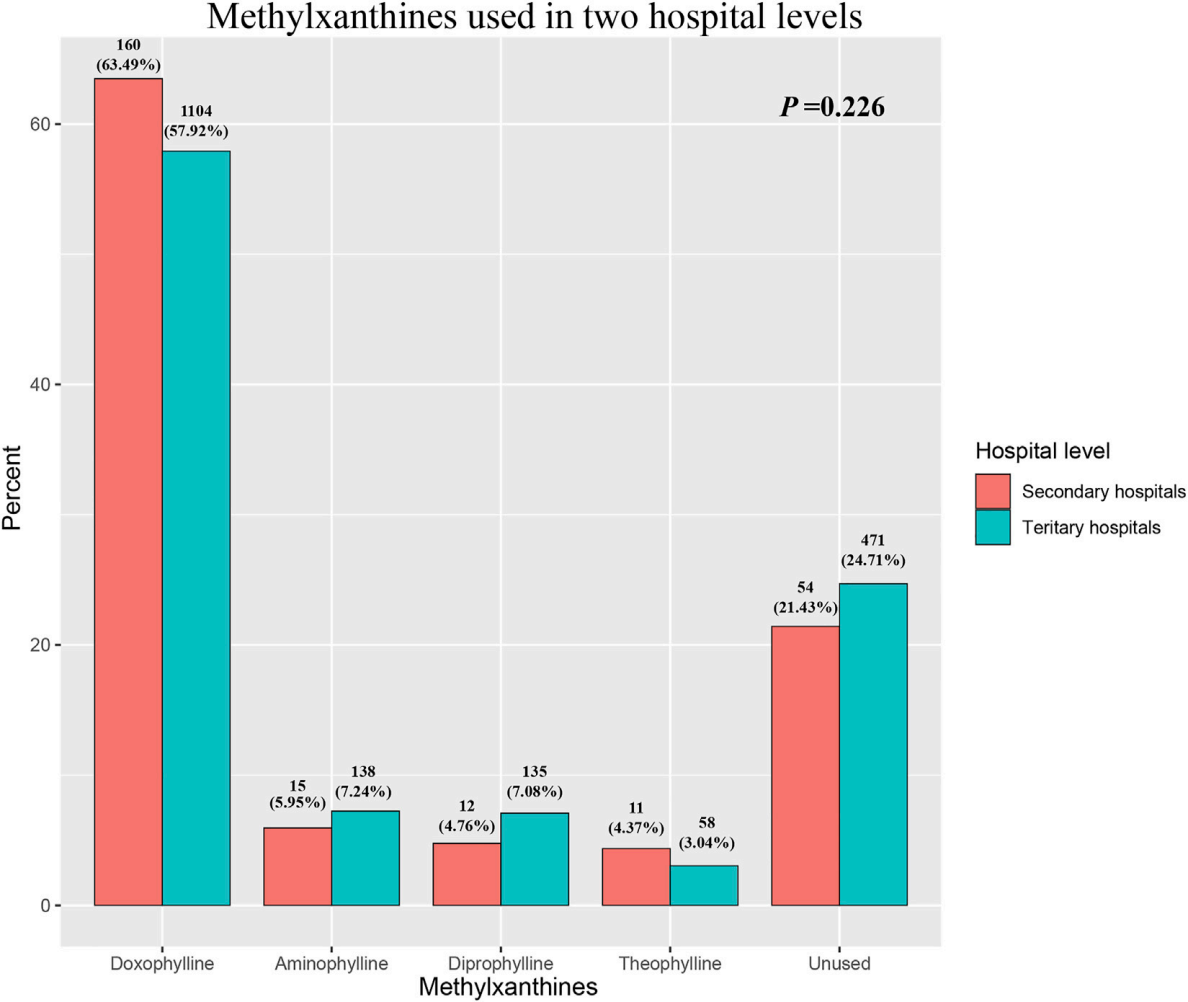
The distribution of methylxanthines used in two hospital levels.

The groups were imbalanced for the smoking history, AECOPD symptoms, including cough, wheezing, and dyspnea, comorbid cardiovascular disease, post-dose FEV1/FVC, GOLD stage, and use of bronchodilators, antibiotics and corticosteroids. Patients who received methylxanthines seemed to have severe AECOPD; they had severe cough (*p* = 0.002), dyspnea (*p* < 0.001), and wheezing (*p* = 0.010) at admission. Patients in the treatment group had a history of smoking (*p* = 0.029) and comorbid cardiovascular disease (*p* = 0.034). Spirometry test results showed that patients in the treatment group had a severe deterioration of lung function, as characterized by the FEV1/FVC (*p* < 0.001) and GOLD stage (*p* < 0.001). The CAT score in the treatment group (median: 20, IQR: 15–25) was slightly higher than that in the control group (median: 19, IQR: 15–23); however, the difference was statistically insignificant (*p* = 0.075). Among all AECOPD patients, 1,562 (74.8%) received either short-acting muscarinic antagonists (SAMA) or short-acting beta-antagonists (SABA). Further, 1,233 of the 1,563 (78.9%) patients in the treatment group received either SAMA or SABA; this proportion was significantly higher than that in the control group (329, 62.7%) (*p* < 0.001). Nearly half of the enrolled AECOPD patients received SABA during hospitalization, and there was no significant difference between groups; however, more patients in the treatment group (591, 37.8%) received SAMA than in the control group (135, 25.7%) (*p* < 0.001). Corticosteroids were commonly used for patients in the treatment group, regardless of the administration route. Methylxanthines were commonly used in both secondary and tertiary hospitals, and doxophylline was the most popular methylxanthine drug (63.49% in secondary hospitals and 57.92% in tertiary hospitals; Figure 2). The type of methylxanthine use was not significantly different between the two hospital levels (*p* = 0.226).

After PS matching, a total of 966 patients were divided equally into two groups, and all baseline characteristics were balanced. A total of 772 (79.9%) patients in the matched cohort were male, and the median age was 70 years (IQR: 64–76).

### Clinical Outcomes During Hospitalization

After PS matching, the median LOS for both the treatment and control groups was approximately 8 days (IQR: 7–11), without any significant difference (*p* = 0.730). However, a significant decrease was observed in the CAT score between the groups (treatment: median, 8; IQR: 4–12 *vs.* control: median: 6, IQR: 2–10; *p* < 0.001). The CAT score at discharge was also lower in the treatment group (median: 10, IQR: 8-14) than the control group (median 12, IQR: 8–16) (*p* < 0.001). There was no significant difference in the total cost, ICU admission, and in-hospital mortality between the groups (Table 3).

**TABLE 3:** Outcomes of hospitalized AECOPD patients treated with or without methylxanthines.

	Before PSM		*p*-Value	After PSM		*p*-Value
	Treatment group (*n* = 1,563)	Control group (*n* = 525)		Treatment group (*n* = 483)	Control group (*n* = 483)	
Length of stay, days	9 (7–12)	8 (7–11)	0.061	8 (7–11)	8 (7–11)	0.730
CAT score at discharge	11 (8-15)	12 (8-16)	<0.001*	10 (8-14)	12 (8-16)	<0.001*
Change in CAT score	8 (4–12)	6 (2–10)	<0.001*	8 (4–12)	6 (2–10)	<0.001*
Total cost, US dollars	1,323 (971–1850)	1,259 (985–1791)	0.227	1,267 (927–1738)	1,262 (994–1793)	0.280
Oxygen therapy	1,232 (78.8)	390 (74.3)	0.031*	376 (77.8)	367 (76.0)	0.492
NPPV	51 (3.3)	6 (1.1)	0.010*	7 (1.4)	6 (1.2)	0.780
IPPV	1 (0.1)	0 (0)	1.000	0	0	1.000
ICU admission	11 (0.7)	3 (0.6)	1.000	2 (0.4)	3 (0.6)	1.000
In-hospital mortality	2 (0.1)	0	1.000	1 (0.2)	0	1.000
30-days follow-up available	921 (58.9)	325 (61.9)	0.229	273 (56.5)	300 (62.1)	0.077
30-days CAT score	11 (8–15)	12 (8–17)	0.012*	10 (8–14)	12 (8–17)	0.006*
30-days all-cause readmission	34 (3.7)	2 (0.6)	0.003*	5 (1.8)	1 (0.3)	0.108
30-days AECOPD readmission	24 (2.6)	1 (0.3)	0.009*	4 (1.5)	0	0.051

Data are presented as n (%) or median (interquartile range). Abbreviations: AECOPD, acute exacerbation of chronic obstructive pulmonary disease; CAT, COPD assessment test; ICU, intensive care unit; IPPV, invasive positive pressure ventilation; NPPV, non-invasive positive pressure ventilation; PSM, propensity score matching.

In the univariable Cox regression analysis, the use of methylxanthines was not significantly associated with the LOS (HR 0.97, 95% CI: 0.86–1.10, *p* = 0.644). After adjusting for the age, sex, BMI, sputum history, wheezing history, mMRC score, CAT score, comorbid cardiovascular and cerebrovascular diseases, history of hospitalization in the previous year, post-dose FEV1/FVC, use of antibiotics, and use of oral and transvenous corticosteroids, the use of methylxanthines was not significantly associated with the LOS (adjusted HR 1.02, 95% CI: 0.89–1.16, *p* = 0.783) (Table 4).

**TABLE 4:** Cox proportional hazards regression model for length of stay.

Variables	Univariable analysis		Multivariable analysis	
	HR (95% CIs)	*p* Value	aHR (95% CIs)	*p* Value
Use of methylxanthines	0.97 (0.86–1.10)	0.644	1.02 (0.89–1.16)	0.783
Age, years	0.99 (0.98–1.00)	0.003*	0.99 (0.99–1.00)	0.155
Male (*vs.* female)	0.81 (0.69–0.95)	0.009*	0.80 (0.68–0.94)	0.008*
Race, han (*vs.* others)	0.87 (0.64–1.17)	0.358	-	-
Smoking history (*vs.* never smokers)	-	-	-	-
Current smokers	0.94 (0.80–1.11)	0.477	-	-
Former smokers	0.87 (0.80–1.01)	0.068	-	-
BMI, kg/m^2^	1.00 (1.00–1.00)	<0.001*	1.00 (1.00–1.00)	<0.001*
Cough (*vs.* none)	-	-	-	-
Morning only	0.82 (0.55–1.23)	0.332	-	
Episodes during day	0.70 (0.47–1.05)	0.088	-	
Nearly continuous	0.67 (0.43–1.04)	0.074	-	
Wheezing (yes *vs.* no)	0.79 (0.67–0.94)	0.008*	0.95 (0.79–1.14)	0.567
Sputum (*vs.* < 10 ml)	-	-	-	-
10–50 ml	0.89 (0.71–1.11)	0.288	0.92 (0.73–1.16)	0.486
50–100 ml	0.77 (0.61–0.97)	0.024*	0.84 (0.66–1.08)	0.178
>100 ml	0.80 (0.55–1.16)	0.244	0.89 (0.61–1.31)	0.568
mMRC score	0.88 (0.82–0.94)	<0.001*	0.92 (0.85–1.00)	0.043*
CAT score	0.98 (0.97–0.99)	<0.001*	1.00 (0.99–1.01)	0.681
Comorbidities	-	-	-	-
Cardiovascular disease (yes *vs.* no)	0.72 (0.64–0.82)	<0.001*	0.77 (0.67–0.88)	<0.001*
Cerebrovascular disease (yes *vs.* no)	0.64 (0.48–0.86)	0.003*	0.68 (0.51–0.92)	0.011*
Previous year admission (*vs.* None)	-	-	-	-
Once	0.86 (0.74–1.00)	0.050*	0.90 (0.77–1.05)	0.170
Twice	0.85 (0.71–1.02)	0.084	0.99 (0.82–1.19)	0.920
More than twice	0.81 (0.62–1.05)	0.106	0.93 (0.72–1.22)	0.612
Post-dose FEV1, l	1.02 (0.99–1.04)	0.149	-	-
Post-dose FEV1/FVC	1.91 (1.09–3.37)	0.025*	1.93 (1.06–3.54)	0.032*
Use of antibiotics	0.68 (0.56-0.81)	<0.001*	0.69 (0.57–0.84)	<0.001*
Eosinophil percent (≥2% *vs.* <2%)	1.03 (0.90–1.17)	0.696	-	-
Use of SAMA	0.93 (0.80–1.08)	0.332	-	-
Use of SABA	0.95 (0.84–1.08)	0.451	-	-
Use of inhaled CS	0.89 (0.75–1.07)	0.226	-	-
Use of oral CS	0.61 (0.40–0.95)	0.027*	0.66 (0.43–1.04)	0.073
Use of transvenous CS	0.77 (0.67–0.89)	0.001*	0.87 (0.75–1.02)	0.085
Use of nebulized CS	1.01 (0.89–1.14)	0.921	-	-

Variables increase the length of stay if HR < 1, whereas variables reduce the length of stay if HR > 1. Abbreviations: aHR, adjusted hazard ratio; BMI, body mass index; CIs, confidence intervals; CS, corticosteroid; CAT, COPD assessment test; Eos, eosinophil; FEV1, forced expiratory volume in one second; FVC, forced vital capacity; HR, hazard ratio; mMRC, modified british medical research council; SAMA, short-acting muscarinic antagonist; SABA, short-acting beta-antagonists.

### Clinical Outcomes up Till the 30-days Follow-up Visit

Among the 2088 enrolled patients, 30-days follow-up data was available for 1,246 (59.7%) patients after discharge. Baseline characteristics were similar between patients with and without 30-days follow-up data (Supplementary Table S1). After PS matching, 5 of 273 (1.8%) patients were readmitted to the hospital within 30 days with any cause in the treatment group. Only 1 of 300 (0.3%) patients were readmitted to the hospital within 30 days with any cause in the control group, and there was no significant difference between these groups (*p* = 0.107). Four of 273 (1.5%) patients were readmitted to the hospital because of AECOPD within 30 days in the treatment group, and there were no cases in the control group that had 30-days AECOPD-related readmission. However, there was no significant difference in AECOPD-related readmissions between the two groups (*p* = 0.051). The CAT score of the treatment group (median, 10, IQR: 8–14) at 30 days after discharge was still significantly lower than that of the control group (median, 12, IQR: 8–17, *p* = 0.006).

### Subgroup Analysis

**FIGURE 3:**
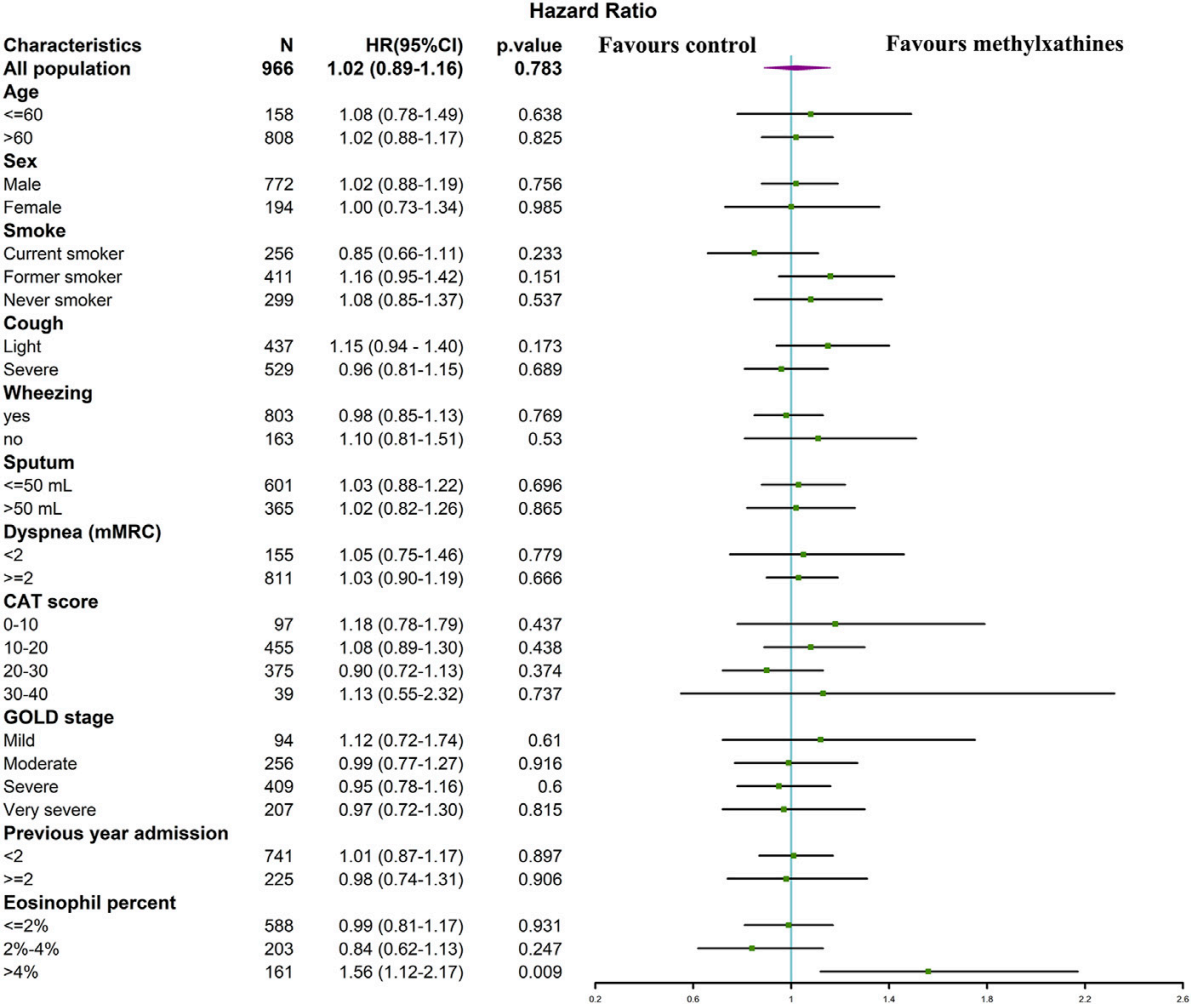
The association between LOS and methylxanthines use in subgroups of matched cohort. Variables increase the length of stay if HR < 1, whereas variables reduce the length of stay if HR > 1.

In the subgroup analysis of the matched cohort for LOS, the association between the use of methylxanthines and LOS remained insignificant in AECOPD patients across different categories of the age, sex, smoking history, severity of AECOPD symptoms, CAT score, GOLD stage, and history of hospitalization in the previous year. The use of methylxanthines was significantly associated with a short LOS only in patients with blood eosinophil counts >4% (adjusted HR: 1.56, 95% CI: 1.12–2.17) (Figure 3).

## Discussion

To the best of our knowledge, this is the first multicenter study that describes methylxanthine use in China and investigates the efficacy of methylxanthine treatment for hospitalized AECOPD patients. In our study, we found that a large number of hospitalized AECOPD patients around China received methylxanthine treatment. Among the commonly used methylxanthine drugs, doxophylline was the most common in both secondary and tertiary hospitals. Patients receiving add-on methylxanthine treatment tend to have severe disease as indicated by severe symptoms, spirometry tests, and comorbid diseases. We performed a 1:1 PS matching analysis to estimate the efficacy of methylxanthines. The matched cohort analysis showed significant symptomatic relief in patients who received methylxanthines during hospitalization. However, we did not find significant differences in the LOS, total cost of hospitalization, ICU admission, and in-hospital mortality. To investigate the subgroup of AECOPD patients who could benefit from methylxanthine treatment, we performed a subgroup analysis between methylxanthine treatment and the LOS in a subset of patients across categories of variables of interest. Interestingly, we found that patients with higher blood eosinophil counts may be associated with a shorter LOS and could benefit from methylxanthine treatment.

The methylxanthine family of drugs includes theophylline, aminophylline, diprophylline, and doxophylline. Theophylline is a classic type of methylxanthine, and several molecular mechanisms have been proposed for its therapeutic effect. It may inhibit phosphodiesterase (PDE) non-selectively, be antagonistic to adenosine receptors, and activate certain histone acetylases (Barnes, 2013). Recently, researchers have investigated the potential use of low-dose theophylline to reverse corticosteroid resistance (Cosio et al., 2004). Several randomized control trials using theophylline as an add-on therapy to corticosteroids for stable COPD patients have been conducted. However, the efficacy of low-dose theophylline add-on therapy is limited (Devereux et al., 2018; Devereux et al., 2019; Jenkins et al., 2020). As for management of acute exacerbations, an early meta-analysis revealed that treatment with theophylline or aminophylline did not improve the FEV1 during hospitalization and LOS. Inversely, some side effects such as nausea and vomiting were observed in patients in the treatment group (Barr et al., 2003). As a result, the GOLD report did not recommend theophylline or aminophylline for the management of AECOPD patients. Consistent with guidelines, our study found limited use of theophylline or aminophylline at both secondary and tertiary hospitals.

Doxophylline is another xanthine derivative with anti-inflammatory and bronchodilator effects. The molecular mechanisms of doxophylline are partially different from those of theophylline. It has decreased affinity toward adenosine A1 and A2 receptors, which may contribute to its better safety profile (Matera et al., 2017; Cazzola and Matera, 2020). Studies comparing the safety profile of doxophylline with other types of methylxanthines revealed fewer side effects than those with theophylline and aminophylline in COPD patients. In addition, it seems that doxophylline has significant efficacy in improving lung function, similar to the therapeutic effects of theophylline and aminophylline (Cazzola et al., 2018). Furthermore, although doxophylline has no significant effect on activation of certain histone acetylases, a recent study using a combination of doxophylline and dexamethasone in a murine model of lung inflammation indicated a corticosteroid-sparing effect at a low dose (Riffo-Vasquez et al., 2018). Thus, because of its good safety profile and promising efficacy, doxophylline was the most common drug used among the hospitalized AECOPD patients receiving methylxanthine treatment in our study. Similar to our study findings, methylxanthines in COPD patients are also widely used in Korea. In 2013, 63.4% of patients with COPD prescribed methylxanthines in Korea. However, the researchers did not analyze the type of methylxanthine and the disease stage for COPD patients (Lee et al., 2017).

In our study, we also designed a PS-matched cohort to investigate the efficacy of methylxanthines in real-world conditions. Unlike the results of a previous meta-analysis that indicated a non-significant change in symptom scores for theophylline and aminophylline treatment in AECOPD patients (Barr et al., 2003), we found significant symptomatic relief in patients who were administered methylxanthine treatment, as assessed by the CAT score. However, these patients did not show a significant reduction in the LOS. Interestingly, further subgroup analysis revealed a significant reduction in LOS for patients with blood eosinophil count >4%.

Eosinophils were recently regarded as an essential biomarker in COPD (Bafadhel et al., 2017). High eosinophils either in blood, sputum or airway were independently associated with frequent exacerbation, worse quality of life, and mortality (Hospers et al., 1999; Hospers et al., 2000; Hastie et al., 2017). It was argued that patients with high eosinophils share some common features with asthma, such as more reversibility to bronchodilators, increased FENO, and better treatment response to corticosteroids (Barnes, 2019). There were several well-designed randomized control trials investigating the role of eosinophils in corticosteroid treatment. For example, when comparing with dual bronchodilator therapy, a randomized clinical trial showed inhaled triple therapy adding beclomethasone dipropionate was associated with a significant reduction of moderate to severe exacerbation in patients with blood eosinophils >2% (Papi et al., 2018). Another hoc analysis of three randomized trials also indicated the exacerbation rate was significantly reduced in the group with blood eosinophils >100 cells/ul, while the efficacy increased at higher blood eosinophil level (Bafadhel et al., 2018). A meta-analysis including 5 randomized control trials indicated 17% reduction in exacerbation rate in patients with blood eosinophils >2% (Cheng, 2018).

Corticosteroids were widely used potent anti-inflammatory agents. They have been reported to induce eosinophil apoptosis through inhibiting the effects of IL-5 and GM-CSF on eosinophil survival and production of cytokines from cytokine-producing cells, such as T cells by means of apoptosis. Interestingly, theophylline, one typical type of methylxanthine, showed some addictive effect on induction of eosinophil apoptosis by functioning as a PDE inhibitor to increase intracellular cyclic adenosine monophosphate on activated eosinophils, the latter can induce apoptosis in eosinophils with some cytokines, such as IL-5 and GM-CSF (Hallsworth et al., 1992; Ohta and Yamashita, 1999). Even though there was no previous studies which investigated the role of methylxanthines in eosinophilic COPD, there was one study about the association between roflumilast, a selective PDE4 inhibitor, and eosinophils which revealed an improved efficacy in high blood eosinophil groups (Martinez et al., 2018). It was also reported that treatment with roflumilast reduced sputum neutrophil and eosinophil numbers by targeting PDE4 (Grootendorst et al., 2007). Thus, it was reasonable to infer that methylxanthines may have a better efficacy in patients with higher blood eosinophils.

An important concern regarding methylxanthine use is the risk of adverse effects. However, the ACURE study lacked data on side effects experienced by patients. Therefore, we could not analyze the side effects that occurred in real-world conditions. There were also other limitations to our study. First, follow-up data after 30 days was not available for all patients in our study. However, we compared the baseline characteristics between those with and those without follow-up data, and we did not find a significant difference, which reduced potential selective bias. Second, since the ACURE study is ongoing we did not have long-term follow-up data. This information will be available in the future and then analyzed. Third, the number of cases with 30-days readmission was limited, and the impact of methylxanthines on short-term readmission needs further study. Finally, for some sub-group analyses, the number of enrolled patients was limited, and further studies for particular AECOPD patients should be conducted.

## Conclusion

This study revealed that methylxanthines, especially doxophylline, are widely used in China for the treatment of AECOPD. Methylxanthines were found to be effective in improving AECOPD symptoms. Higher blood eosinophil levels may be associated with better efficacy of methylxanthine treatment. Further studies are needed to determine the association between eosinophil count and the efficacy of methylxanthines in a large population.

## Data Availability

The original contributions presented in the study are included in the article/Supplementary Materials, further inquiries can be directed to the corresponding authors.
