# Supplementary material for: Methylxanthine Treatment in Patients Hospitalized for Acute Exacerbation of Chronic Obstructive Pulmonary Disease in China: A Real-World Study Using Propensity Score Matching Analysis
Source: Front Pharmacol. 2022 Jan 25;13:802123. doi: 10.3389/fphar.2022.802123 (PMC8821534; doi:10.3389/fphar.2022.802123)
Supplement: Supplementary file 1 [file Table1.docx]

Table S1. Comparation of characteristics of AECOPD patients with or without follow-up data

|  | With follow-up data  (n=1246) | Without follow-up data  (n=842) | p-value |
| --- | --- | --- | --- |
| Age, years | 70 (64–76) | 69 (63–76) | 0.322 |
| Sex, male | 1004 (80.6) | 675 (80.2) | 0.816 |
| Race, han | 1204 (96.6) | 779 (92.5) | <0.001 |
| Smoking history |  |  | 0.039* |
| Current smokers | 377 (30.3) | 212 (25.2) |  |
| Former smokers | 532 (42.7) | 381 (45.2) |  |
| Non smokers | 337 (27.0) | 249 (29.6) |  |
| BMI, kg/m2 | 22.0 (19.5–24.4) | 21.9 (19.2–24.6) | 0.860 |
| Comorbidity |  |  |  |
| Cardiovascular disease | 614 (49.3) | 396 (47.0) | 0.313 |
| Cerebrovascular disease | 61 (4.9) | 42 (5.0) | 0.924 |
| Diabetes mellitus | 117 (9.4) | 74 (8.8) | 0.640 |
| Previous hospitalization within 1 year |  |  | 0.041 |
| 0 | 652 (52.3) | 389 (46.2) |  |
| 1 | 290 (23.3) | 229 (27.2) |  |
| 2 | 194 (15.6) | 149 (17.7) |  |
| 3+ | 110 (8.8) | 75 (8.9) |  |
| Spirometry test |  |  |  |
| Post-dose FEV1 | 0.97 (0.73– 1.35) | 0.98 (0.72–1.37) | 0.606 |
| Post-dose FEV1/FVC | 0.50 (0.42– 0.58) | 0.50 (0.41– 0.59) | 0.957 |
| GOLD stage |  |  | 0.734 |
| I (mild) | 93 (7.5) | 68 (8.1) |  |
| II (moderate) | 335 (26.9) | 241 (28.6) |  |
| III (severe) | 524 (42.1) | 338 (40.1) |  |
| IV (very severe) | 294 (23.6) | 195 (23.2) |  |
| CAT at admission |  |  | 0.389 |
| CAT < 10 | 111 (8.9) | 66 (7.8) |  |
| CAT ≥ 10 | 1135 (91.1) | 776 (92.2) |  |
| Eosinophil count |  |  | 0.146 |
| Eos < 2% n (%) | 746 (60.3) | 471 (57.1) |  |
| Eos ≥ 2% n (%) | 491 (39.7) | 354 (42.9) |  |
| Bronchodilators |  |  |  |
| SAMA | 436 (35.0) | 290 (34.4) | 0.796 |
| SABA | 686 (55.1) | 418 (49.6) | 0.015* |
| Corticosteroids |  |  |  |
| Inhaled CS | 209 (16.8) | 140 (16.6) | 0.930 |
| Oral CS | 44 (3.5) | 26 (3.1) | 0.581 |
| Transvenous CS | 370 (29.7) | 249 (29.6) | 0.952 |
| Nebulized CS | 728 (58.4) | 464 (55.1) | 0.132 |

Data are presented as n (%) or median (interquartile range). Abbreviations: BMI: body mass index; CS: corticosteroid; CAT: COPD assessment test; Eos: eosinophil; FEV1: forced expiratory volume in one second; FVC: forced vital capacity; mMRC: modified British medical research council; SAMA: short-acting muscarinic antagonists; SABA: short-acting beta- antagonists.
